# Prediction of barberry witches’ broom rust disease using artificial intelligence models: a case study in South Khorasan, Iran

**DOI:** 10.1038/s41598-025-97733-6

**Published:** 2025-04-16

**Authors:** Javad Ramezani-Avval Reiabi, Mojtaba Mohammadpoor

**Affiliations:** 1 Plant Protection Management, Agricultural Organization of South Khorasan Province, Birjand, Iran; 2 Computer and Electrical Engineering Department, University of Gonabad, Gonabad, Iran

**Keywords:** Convolutional neural network, Deep learning, Seedless barberry, TensorFlow’s Keras API, Witches’ broom rust, Computational biology and bioinformatics, Plant sciences, Engineering

## Abstract

The South Khorasan Province in Iran is the main producer of seedless barberry, accounting for 98% of the country’s production. This has led to significant economic growth in the region. However, the cultivation of barberry is threatened by the rust fungus Puccinia arrhenatheri, which causes witches’ brooms on Berberis vulgaris L. var. asperma. Our research aims to detect infected leaves containing this fungal pathogen using deep learning (DL)-based artificial intelligence (AI) techniques on an available dataset. We captured healthy and infected barberry foliage images and used conventional laboratory methods to label them. We developed a convolutional neural network (CNN) deep learning model using TensorFlow’s Keras API to detect and classify barberry broom rust disease. A cross-validation technique is used to check the robustness of the proposed model. The results imply that the proposed model successfully distinguished between healthy specimens and those affected by broom rust disease. The model achieved an impressive accuracy rate of 98% in automatically identifying the disease type and its severity. This interdisciplinary research demonstrates the practical application of AI in agriculture, providing timely intervention strategies to protect crop yields and maintain economic viability in the face of plant diseases.

Agriculture plays a crucial role in Iran’s economy, offering substantial opportunities for growth and advancement. The sector currently fulfills approximately 90% of the nation’s food requirements through domestic production. Moreover, it contributes 11.6% to Iran’s gross domestic product (GDP) and employs 18% of the total workforce. Effective crop disease management is essential in agriculture, enhancing yield and quality while mitigating economic and aesthetic losses caused by plant pathogens. Disease outbreaks continually threaten agriculture, resulting in significant economic losses for the country. Efficient detection of plant diseases has long been a formidable challenge in agriculture. Traditional diagnostic methods, such as visual assessment and laboratory analysis, have been the cornerstone of disease identification. However, these methodologies are not without their limitations. They are often labor-intensive, time-consuming, and require specialized expertise, leading to inconsistencies in monitoring and diagnosis. Moreover, the destructive nature of laboratory techniques and their associated costs pose significant hurdles to widespread adoption^1,2^. In contrast, mechanized approaches offer promise in addressing these challenges by providing non-invasive, rapid, and cost-effective solutions. One such approach gaining traction is the use of image processing techniques, which leverage advancements in AI to automate disease detection and classification. By harnessing the power of deep learning (DL) algorithms, these methods offer precision and efficiency in diagnosing plant diseases^3,4^. The applications of DL and machine learning (ML) methods for plant disease detection are experiencing rapid growth. AI is revolutionizing plant disease diagnosis using image analysis techniques. Convolutional neural networks (CNNs), a category of DL algorithms, excel in processing visual data. These networks can thoroughly analyze plant images, identifying subtle differences in color, texture, and shape that indicate the presence of diseases. AI models can classify the severity of diseases and accurately identify specific diseases. This computerized image analysis enables farmers to identify and evaluate diseases easily, with heightened accuracy and efficiency^5,6^.

Advancements in AI are leading to a revolutionary transformation in the field of plant disease management and phytopathological research. AI has proven its ability to detect and diagnose diseases automatically using image recognition techniques. It has been reported that AI achieves accuracies of over 95%, which surpasses the assessment capabilities of human visual perception. AI can revolutionize decision-making in disease prevention and precision management in the field^3^. AI is a groundbreaking concept in computing, completely changing how machines handle tasks that have traditionally relied on human intelligence. It has the power to revolutionize and transform the industry^7,8^. There are various AI models, with CNN standing out as a notable one. CNNs are tailored for image processing and recognition, using convolutional layers to autonomously learn spatial features from input images. Yann LeCun brought CNNs to the scene during the early 1990s, but it wasn’t until the mid-2010s that they gained significant attention for their revolutionary progress in image recognition assignments^3,9,10^.

Multiple studies have consistently demonstrated the success of AI in accurately identifying complex diseases. In a previous study, DL techniques were applied to analyze images and identify Cassava diseases. By utilizing CNN, they achieved a diagnostic accuracy of over 90%, demonstrating the effectiveness of DL in detecting various Cassava diseases. This method not only surpassed conventional methods in terms of accuracy and speed, but it also made it easier to implement these models on mobile devices, enhancing the ease of field diagnosis^11^.

Recent research has shown that using advanced, accurate, and robust models has greatly improved the effectiveness of AI in identifying and diagnosing plant diseases. This improvement is mainly due to the development of new AI capabilities. In 2022, researchers conducted a study to determine the efficacy of visual and automated identification methods in detecting fungal infections in cereal crops, specifically wheat and rice. The study showed that artificial neural networks can accurately identify and categorize disease patterns, such as yellow spots, yellow and brown rust, and brown patches. The classification metrics for these patterns ranged from 0.95 to 0.99^12^.

South Khorasan Province has emerged as a focal point in the battle against barberry diseases, particularly the relentless spread of broom rust, *Puccinia arrhenatheri*, within its barberry orchards over the past two decades (Fig. 1). Broom rust disease, known for its destructive impact on barberry crops, presents a growing threat to agricultural productivity and economic stability in the region. To tackle this challenge, our study examines the use of AI techniques, such as image processing and deep learning algorithms, to detect and classify barberry rust disease. Our goal is to combine cutting-edge technology with agricultural practices to transform disease management strategies in the region. By utilizing AI for quick and accurate diagnosis, we aim to decrease dependence on human expertise, minimize crop damage, preserve resources, and improve the quality of barberry products. Additionally, by taking advantage of mechanized detection methods, we strive to establish a sustainable and resilient agricultural ecosystem that can withstand the difficulties presented by emerging plant diseases.

This study is the first in Iran and elsewhere to accurately and quickly detect barberry broom rust disease using AI tools. We provided a thorough analysis of how AI techniques can be implemented in disease detection, emphasizing their ability to revolutionize agricultural practices.

**Fig. 1:**
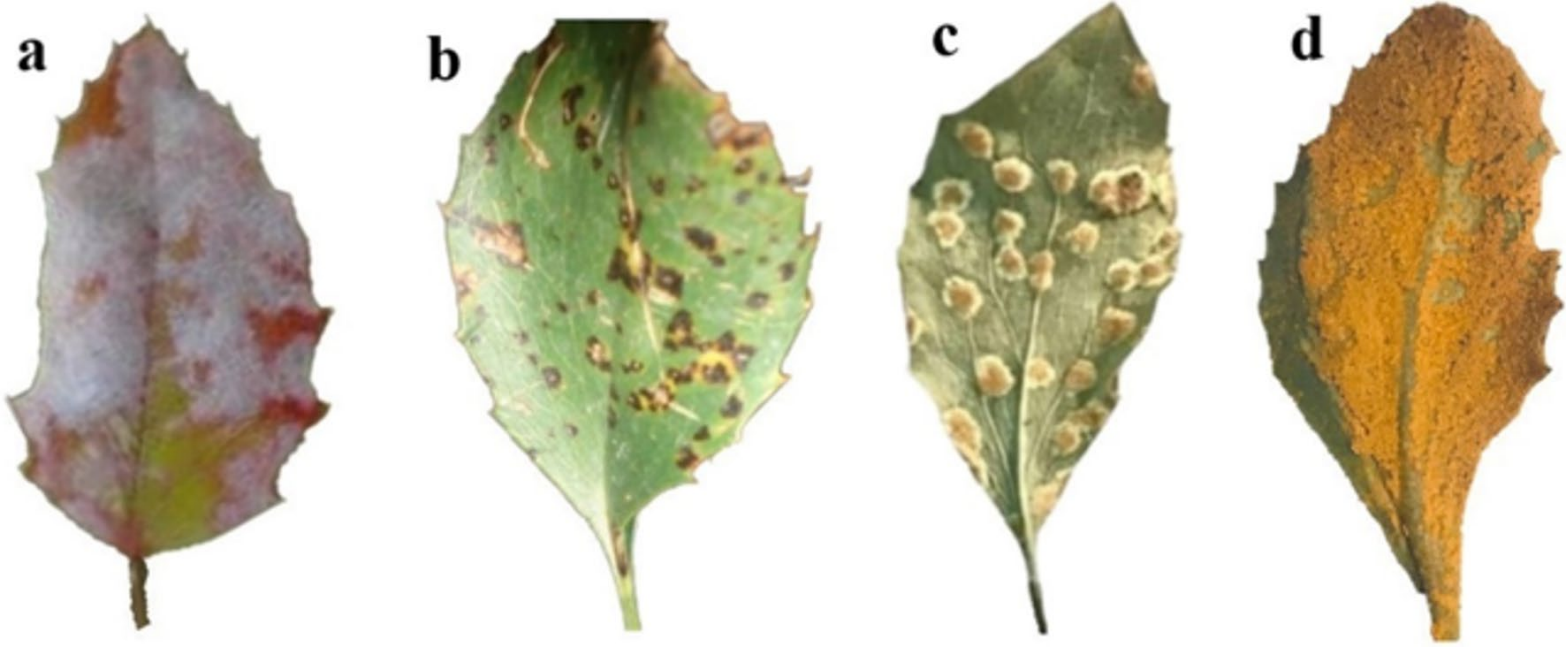
Foliar diseases observed in Barberry orchards in South Khorasan Province of Iran. (**a**) Powdery mildew, a common fungal disease affecting barberry leaves, stems, and sometimes fruits. (**b**) Brown leaf spots, can be attributed to fungal infections, bacterial pathogens, or stress-induced factors. (**c**) Localized symptoms of wheat black or stem rust disease on *Berberis vulgaris* leaves as an alternate host. (**d**) Systemic symptoms of broom rust disease on *Berberis vulgaris* leaves.

and ensure food security. By collaborating across disciplines and utilizing innovative technology, we aim to establish a more effective and sustainable approach to disease management in agriculture.

## Materials and methods

### Data collection

The data collection process was meticulously executed by expert personnel from the Agricultural Organization of South Khorasan, with a keen focus on ensuring the creation of a comprehensive and reliable dataset for the development of a deep learning model to detect barberry rust disease. Recognizing the importance of practical applicability, the data collection took place directly in the field using common smartphone cameras, mirroring the conditions encountered by farmers during routine crop monitoring activities.

A total of 208 images of healthy barberry leaves and 549 images of leaves affected by broom rust disease were captured across various locations within the South Khorasan Province. These images were carefully annotated and classified by field experts to ensure the accuracy and integrity of the dataset. Following the collection phase, during the processing stage, the dataset was divided into training and validation subsets in a k-fold cross-validation method (with k = 5) to facilitate the development and evaluation of the DL model. This division was conducted with careful consideration and the model was run k times. In each round k-1 folds of each data category (equal to 80% of the images) allocated to the training subset and the remaining fold (equal to 20%) to the validation subset.

**Fig. 2:**
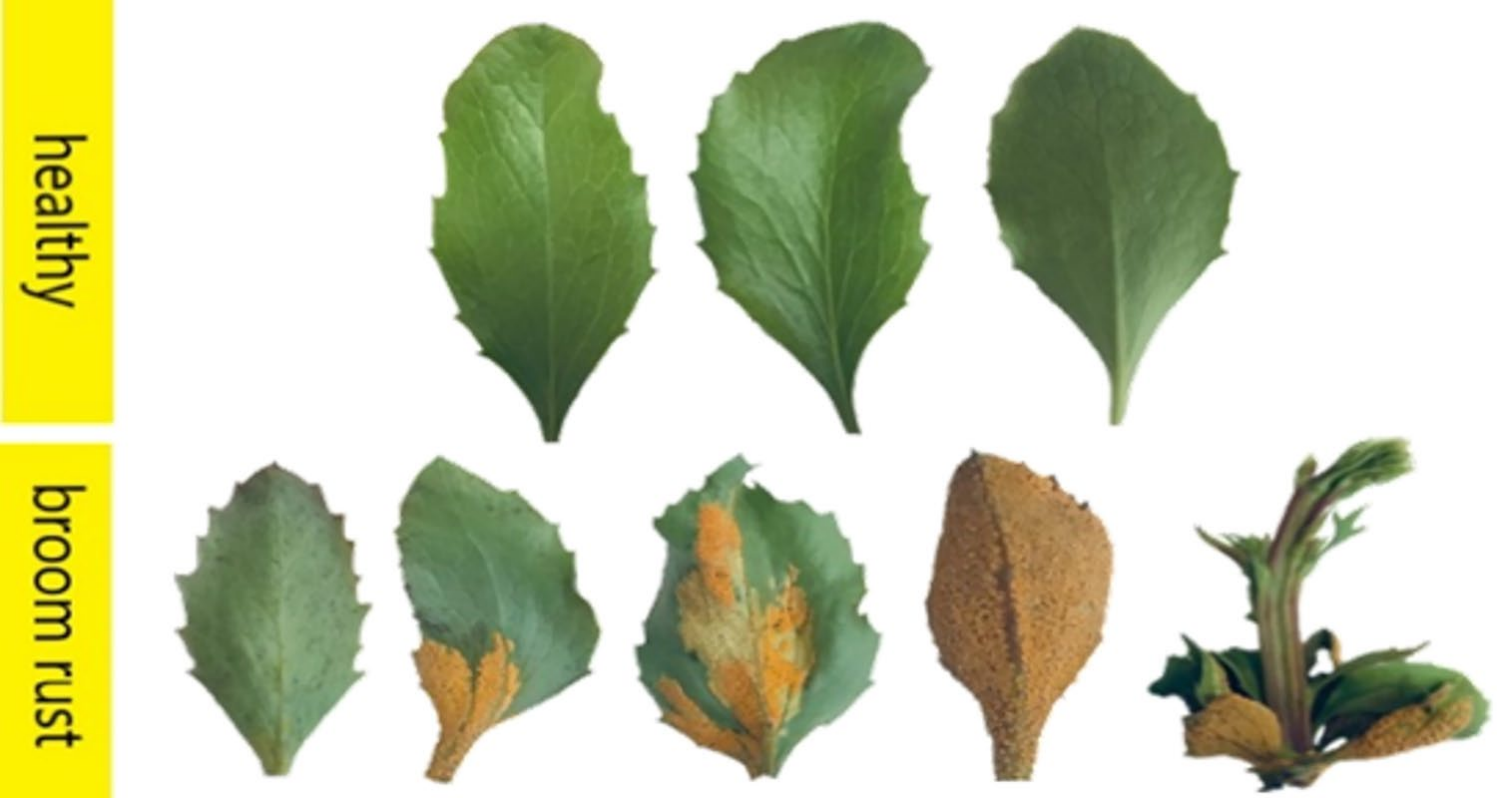
Leaf image examples belonging to healthy and diseased classes of barberry plants.

By incorporating this approach to data division, the dataset provides a solid foundation for the development of a DL model capable of accurately detecting and classifying barberry rust disease. Furthermore, the inclusion of a validation subset enables the assessment of the model’s performance and generalization capabilities, ensuring its reliability and effectiveness in real-world agricultural scenarios. Figure 2 shows examples of leaf images from both healthy and diseased classes of barberry plants.

### Proposed model

**Fig. 3:**
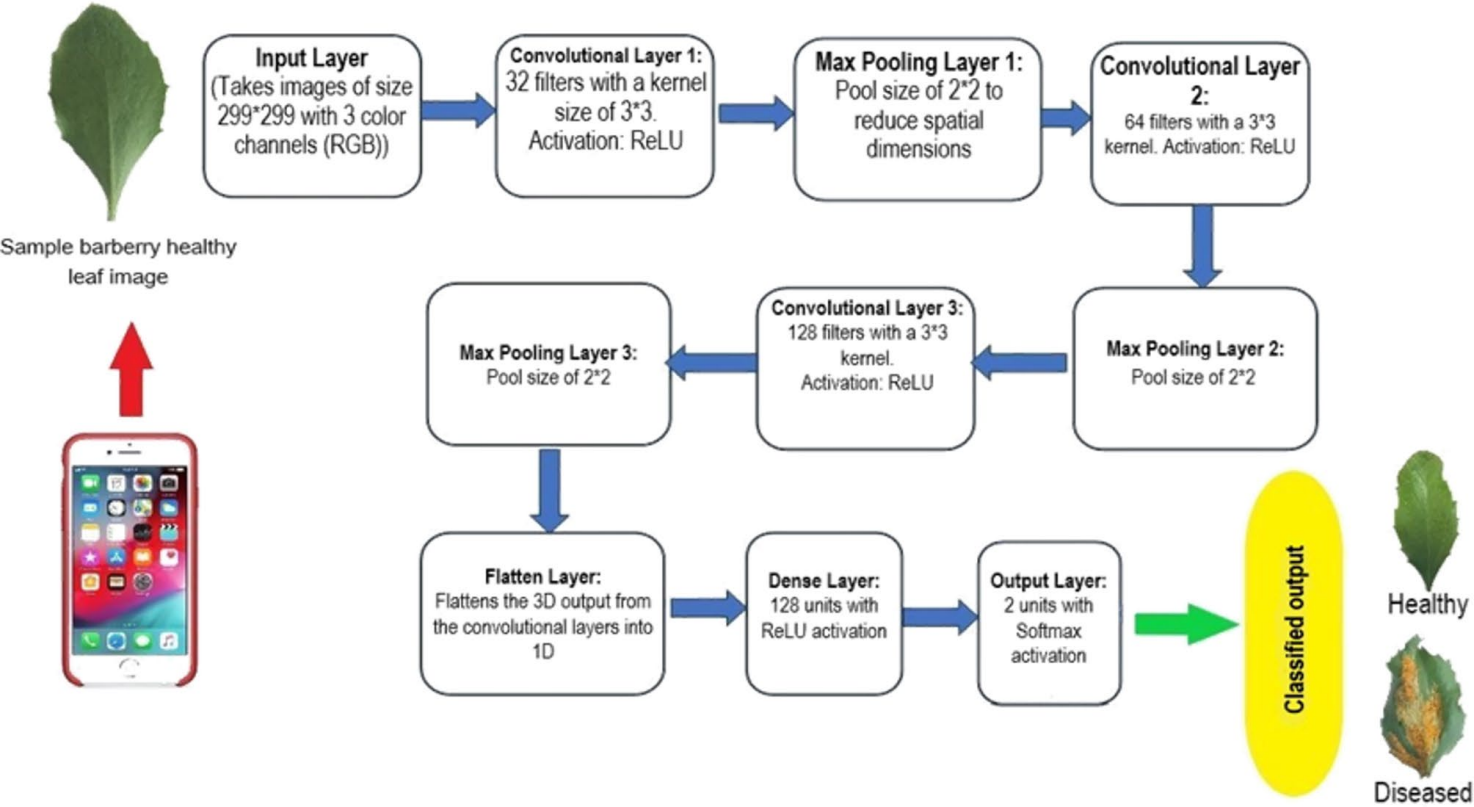
The schematic flowchart of the proposed CNN DL model.

A convolutional neural network (CNN) model has been devised using TensorFlow’s Keras API to address the challenge of detecting and classifying barberry broom rust disease. This model leverages the inherent capabilities of DL to accurately identify disease patterns within barberry leaf images, enabling rapid and efficient diagnosis in agricultural settings. The model architecture includes several convolutional layers that are excellent at capturing detailed spatial features in the input images. These convolutional layers are complemented by max-pooling layers, which down-sample the feature maps to reduce computational complexity and spatial dimensions while preserving important information. Additionally, dense layers are included to enable high-level feature abstraction and classification. The model is compiled using a suitable optimizer, loss function, and evaluation metrics that are specific to the binary classification task. Figure 3 shows the flowchart of the proposed DL model.

In order to evaluate the robustness of the model, cross-validation method is used. In each round, the model is trained on the training dataset (i.e. K-1 fold), and evaluate on remained fold. The overall results shows that proposed model can potentially revolutionize disease detection and management practices in agriculture, specifically in combating the spread of barberry broom rust disease. This is achieved by integrating advanced DL techniques and implementing rigorous evaluation protocols.

### Evaluation standards

There are several metrics to evaluate a classification models. The effectiveness can be evaluated using confusion matrix, accuracy, precision, recall, and F1-score parameters. The metrics are explained bellow and the proposed model is thoroughly evaluated on the validation dataset to determine its performance and ability to generalize^13^.

#### Confusion matrix (CM)

A confusion matrix is a table that summarizes the performance of a classification model. It provides an overview of how well the model performs in classifying data. First, the confusion matrix is computed to visualize the model’s classification results and assess its ability to accurately classify healthy and diseased barberry leaves. This matrix allows for the identification of true positive, true negative, false positive, and false negative predictions, offering valuable insights into the model’s performance across different classes. As shown in Table 1, it provides insights into how well the model is distinguishing between the “Healthy” and “Broom Rust” barberry leaves.

**Table 1:** Confusion matrix explanation.

Classes	Predicted Broom Rust	Predicted Healthy
Actual Broom Rust	TN (True Negative)	FP (False Positive)
Actual Healthy	FN (False Negative)	TP (True Positive)

##### True positive (TP)

The model correctly predicts “Broom Rust” when the actual class is “Broom Rust.”

##### True negative (TN)

The model correctly predicts “Healthy” when the actual class is “Healthy.”

##### False positive (FP)

The model incorrectly predicts “Broom Rust” when the actual class is “Healthy” (Type I error).

##### False negative (FN)

The model incorrectly predicts “Healthy” when the actual class is “Broom Rust” (Type II error).

By examining the values in the confusion matrix, we can assess the model’s performance and understand its tendencies:

A high value in the TP cell indicates that the model accurately identifies instances of “Broom Rust. A high value in the TN cell indicates that the model is effective in accurately identifying instances of “Healthy.”

A high value in the FP cell indicates that the model occasionally misidentifies instances labeled as “Healthy” as “Broom Rust.”

A high value in the FN cell indicates that the model occasionally incorrectly identifies instances of “Broom Rust” as “Healthy.”

Understanding the confusion matrix is crucial for assessing the model’s performance in distinguishing between the two classes and pinpointing areas for possible improvements.

In addition, it is possible to generate a detailed classification report that includes metrics such as precision, recall, and F1-score for each class. These metrics offer a thorough assessment of the model’s performance by considering both the occurrence of false positives and false negatives. This comprehensive assessment provides a deep understanding of the model’s predictive abilities.

#### Accuracy

Accuracy is a frequently used metric for evaluating classification tasks. It calculates the ratio between the number of correctly labeled instances and the total number of examples. Mathematically, accuracy can be calculated using a specific equation.

1 $$\:Accuracy=\frac{Number\:of\:Correct\:Predictions\:}{Total\:Number\:of\:Predictions\:}$$

#### Precision

The accuracy of positive predictions is determined by calculating the ratio of true positive predictions (examples correctly predicted as positive) to all examples predicted as positive. Precision measures the ratio of true positive predictions to the total number of positive predictions made by the model. It can be calculated by dividing the number of true positives by the sum of true and false positives. A high precision value indicates that the model makes fewer false positive predictions. Mathematically, precision can be expressed as follows.

2 $$\:Precision=\frac{TruePositives\:}{TruePositives\:+\:FalsePositives\:}$$

#### Recall

Recall, also known as sensitivity or true positive rate, quantifies the model’s proficiency in accurately detecting positive examples. It is calculated by dividing the number of true positive predictions by the total number of true positive examples. A high recall indicates that the model captures a high proportion of actual positive examples. Mathematically, recall can be expressed by the following equation.

3 $$\:Recall=\frac{TruePositives\:}{TruePositives+FalseNegatives\:}$$

#### F1 score

The F1 score is a metric that considers false positives and false negatives. It is calculated as the harmonic mean of precision and recall. The F1-score reaches its best value at 1 and worst value at 0. Mathematically, the F1 score can be expressed using the following formula.

4 $$\:F1\:Score=\frac{2\:.\:Precision\:.\:Recall}{Precision\:+\:Recall\:}$$

Furthermore, the Receiver Operating Characteristic (ROC) curve can be graphed, and the Area Under the Curve (AUC) score can be calculated. This curve represents the connection between the true positive rate and false positive rate of the model at different threshold settings. It offers valuable insights into the trade-off between these two factors. It offers valuable insights into the model’s capacity to discriminate and perform effectively across different decision thresholds. The proposed model’s effectiveness and generalization capabilities can be thoroughly assessed by utilizing these evaluation metrics. This assessment enables informed decisions regarding the model’s deployment in real-world agricultural settings. The model’s reliability and suitability for practical applications in disease detection and management are established by employing robust evaluation methodologies. This paves the way for enhanced agricultural productivity and sustainability.

## Results and discussion

In order to evaluate the proposed method and enhance the robustness of the results and improve model reliability, cross-validation method is applied. The images of each category is divided into five folds. The model is ran five times, training by four of folds in turn, and evaluate by the remained one.

In addition, since we face an imbalance number of images in two classes, a class weighting technique is performed during training the model to prevent it biasing toward the majority class. In this technique, by assigning higher weights to the minority class (i.e. healthy leaves), the model is encouraged to pay more attention to the minority class during training, thereby improving its ability to correctly classify minority class samples.

### Average confusion matrix

Confusion matrix is a table that provides a concise overview of how well a model accurately classifies data. It displays the number of true positives, true negatives, false positives, and false negatives. By analyzing the information provided in the CM, important metrics such as precision and recall can be calculated. In case of using cross-validation, average CM can be reported.

**Fig. 4:**
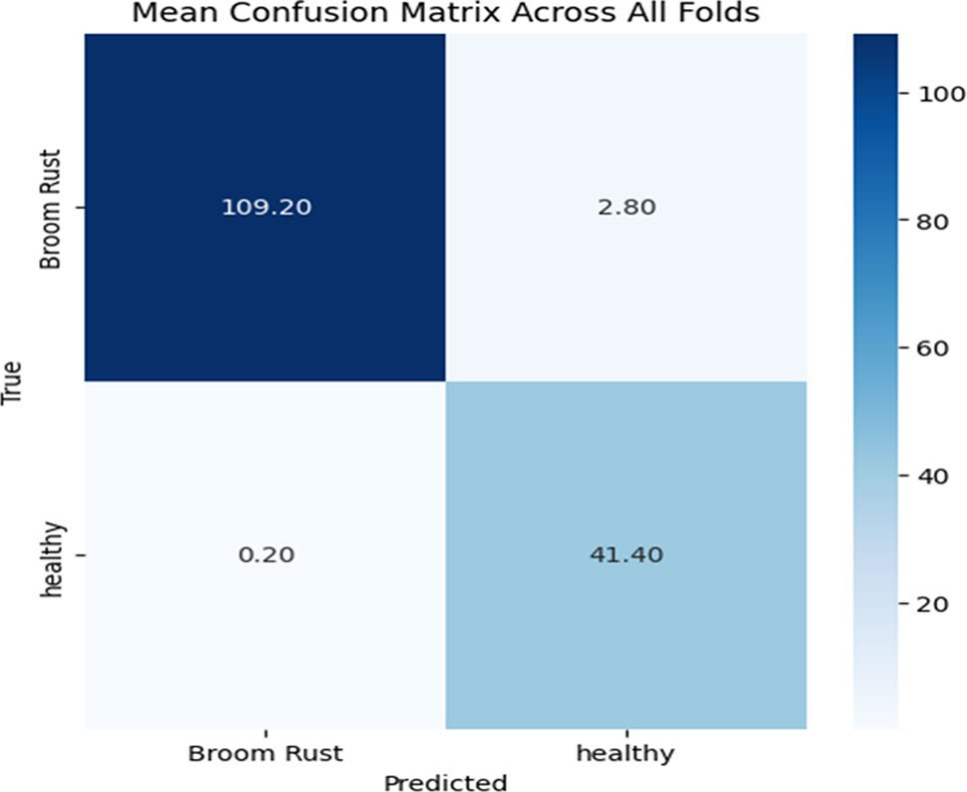
Average confusion matrix.

Figure 4 shows the Average CM generated by applying the trained model to the validation portions of the dataset. There are an average of total of 41 healthy images and 110 affected images in each fold. As shown, very few images of each category are predicted incorrectly. Broom Rust False Positive (Healthy leaves that incorrectly classified as Broom Rust) is equal to two, and Healthy False Positive (Broom Rust cases that incorrectly classified as healthy) is negligible, in average.

### Evaluation metrics

Table 2 shows the detailed average classification reports of the model.

**Table 2:** Average classification reports of the model.

Average Validation Accuracy across 5 folds	0.9805
Average F1 Score across 5 folds	0.9647
Average Precision across 5 folds	0.9365
Average Recall across 5 folds	0.995
Average AUC across 5 folds	0.9962

As it shown, on average, the proposed model correctly predicted the class labels 98.05% of the time across the 5 folds of cross-validation, suggesting that the model is performing exceptionally well on the validation data.

An F1 score of 0.9647 is high enough, indicating that the model has a strong balance between precision and recall. This suggests that the model is not only accurate but also robust in handling both false positives and false negatives.

A precision of 0.9365 means that 93.65% of the positive predictions made by the model are correct, indicating that the model has a low rate of false positives.

A recall of 0.9950 means that the model correctly identified 99.5% of the actual positive cases, indicating that the model has a very low rate of false negatives.

The model achieved an impressive ROC AUC score of 0.9962, which confirms its accuracy in distinguishing between “Broom Rust” and “Healthy” leaves. This indicates that the model is highly effective in classifying instances of disease and health with great confidence. The robust ROC AUC score of 0.9962 further reinforces the results from the classification report, offering additional evidence of the model’s outstanding performance in detecting barberry broom rust disease.

**Fig. 5:**
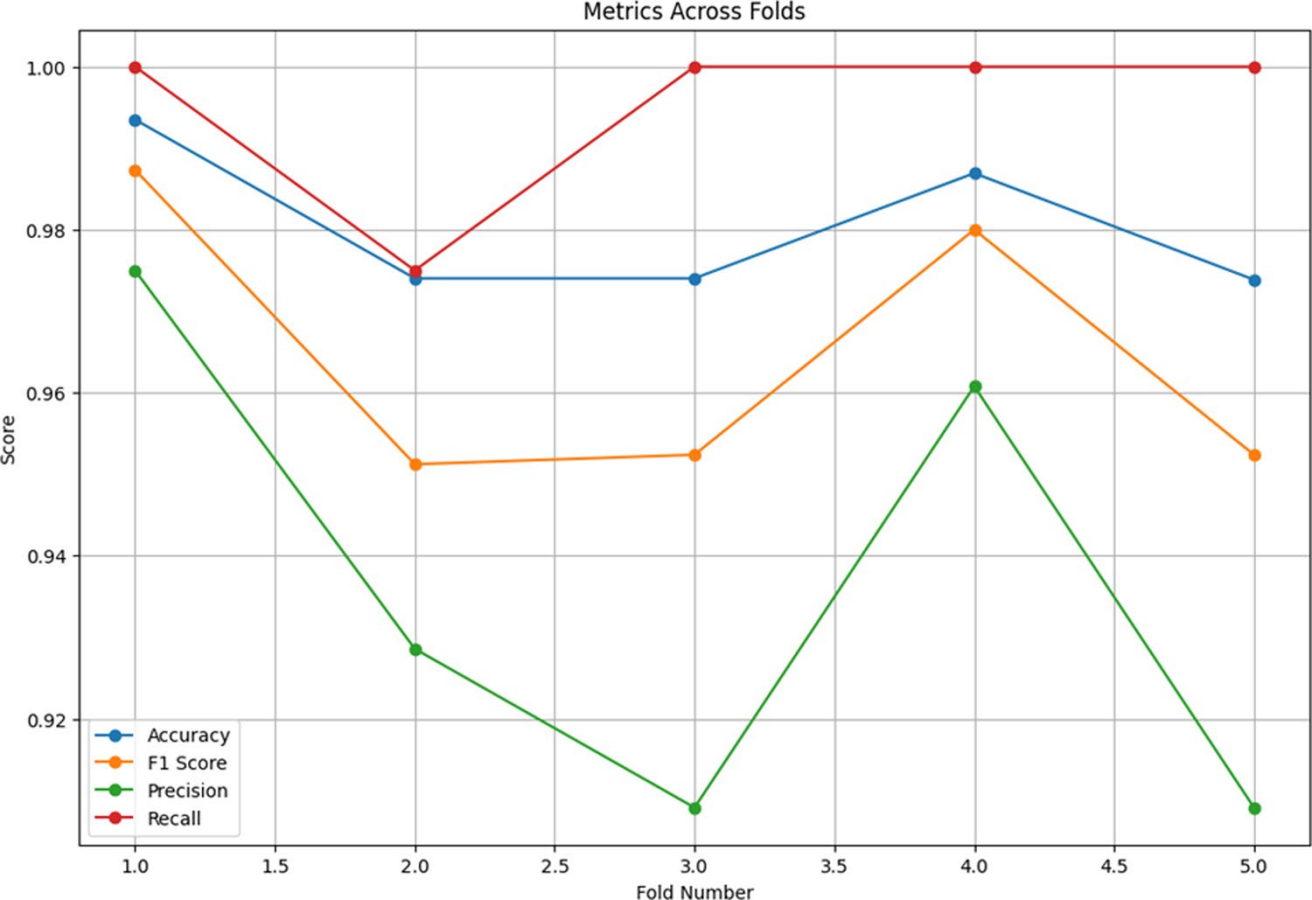
Evaluation metrics across folds.

Figure 5 is showing evaluation metrics in each fold. As it shown, almost all metrics have experience acceptable values in each fold.

**Fig. 6:**
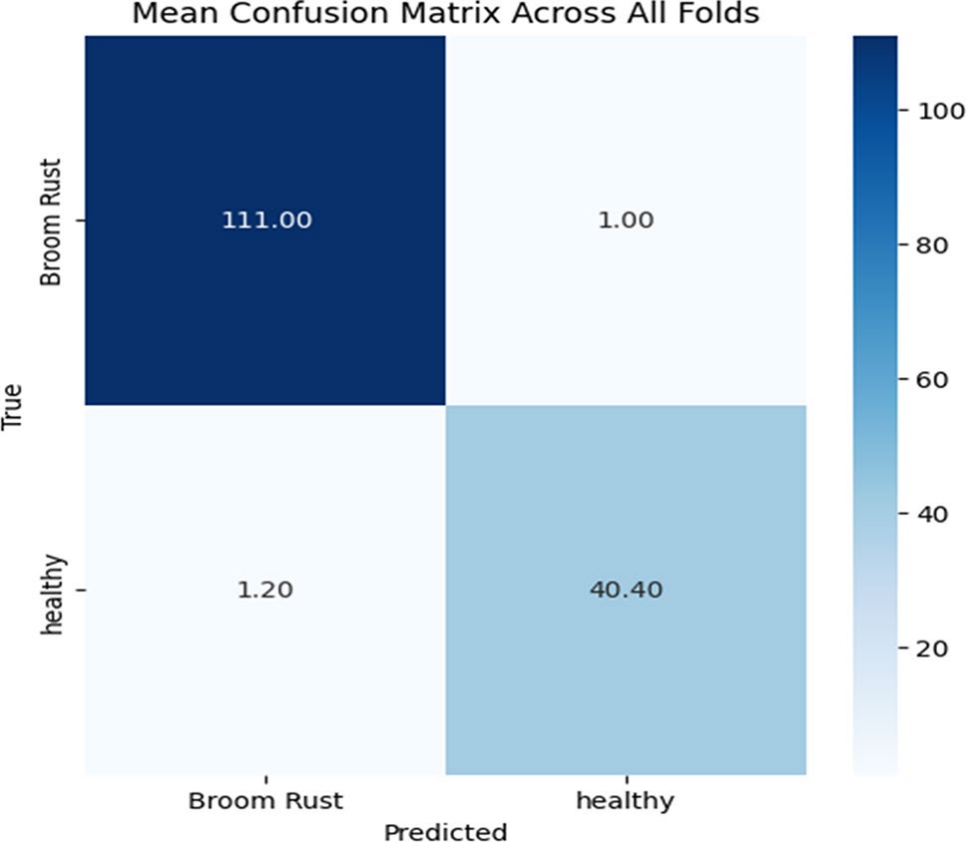
Average confusion matrix of VGG16 model.

**Fig. 7:**
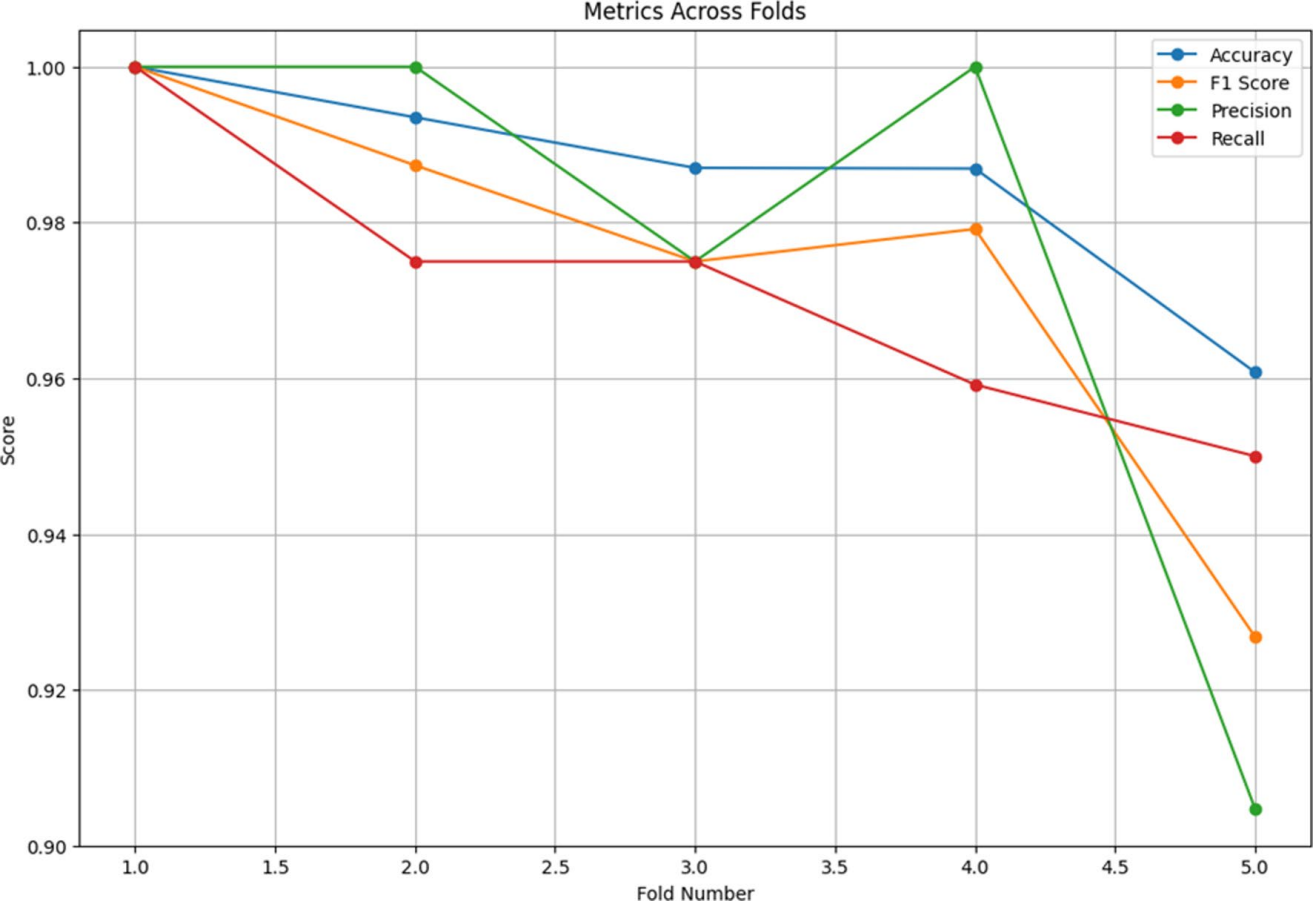
Evaluation metrics across folds.

In order to compare the proposed method by state-of-the-art models, some pre-trained models are performed in the same conditions. In this regard, VGG16 and MobileNetV2 models with pre-trained weights form ‘imagenet’ is used. Figures 6 and 7 are showing the Average CM, and evaluation metrics across folds of the VGG16 model, respectively.

**Fig. 8:**
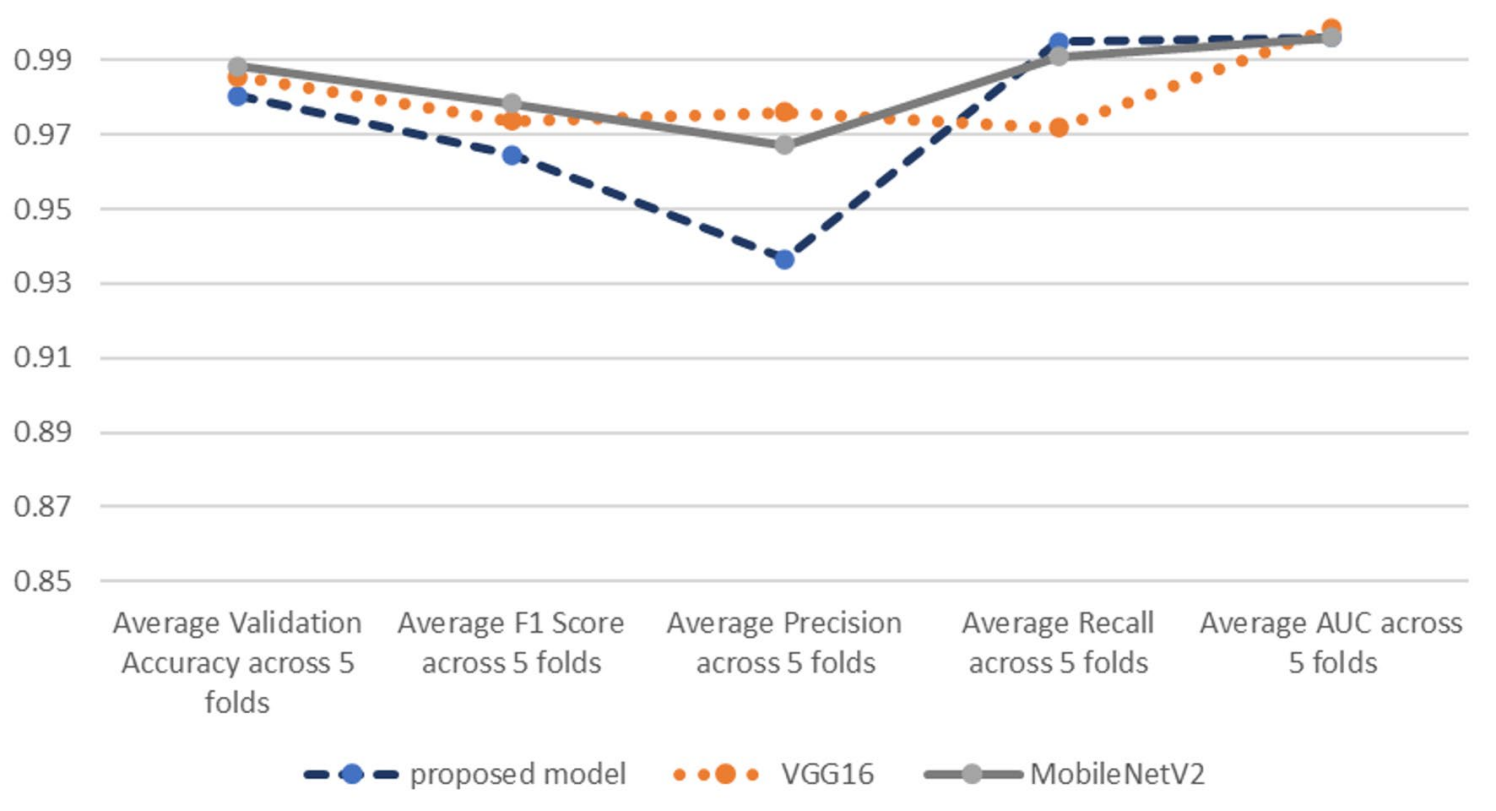
Average classification reports of proposed model, VGG16 and MobileNetV2 models.

Table 3 is showing the average classification reports of VGG16 and MobileNetV2 models, and Fig. 8 compares them by the proposed model.

**Table 3:** Average classification reports of VGG16 and MobileNetV2 models.

metric	VGG16	MobileNetV2
Average Validation Accuracy across 5 folds	0.9856	0.9883
Average F1 Score across 5 folds	0.9737	0.9784
Average Precision across 5 folds	0.9760	0.9671
Average Recall across 5 folds	0.9718	0.9909
Average AUC across 5 folds	0.9986	0.9961

By comparing the results of proposed model with VGG16 and MobileNetV2, despite being a simpler sequential model, which is an advantage especially when computational resources are limited, it has a competitive performance and performs very close to VGG16 and MobileNetV2 in most metrics.

## Conclusion and future works

Substantial advancements have been achieved in the field of agricultural disease management through the development and assessment of the DL model for detecting barberry broom rust disease. The model has been thoroughly evaluated and has proven its capability to accurately distinguish between diseased and healthy barberry leaves, as shown by the classification report. The model achieved exceptional precision, recall, and F1-score in detecting broom rust leaves, with a flawless score of 0.98 across all metrics. This demonstrates its ability to accurately and reliably identify instances of the disease. Furthermore, the model performed admirably in classifying healthy leaves, achieving a precision, recall, and F1-score of more than 0.97. Moreover, the model’s impressive accuracy rate of 98% demonstrates its effectiveness in distinguishing between broom rust and healthy leaves. This highlights its potential for practical use in agricultural settings. By combining advanced DL techniques with rigorous evaluation methods, the proposed model presents a promising solution for the early detection and effective management of barberry broom rust disease. The model assists farmers in the South Khorasan Province and beyond by enabling them to detect disease outbreaks early. This leads to enhanced agricultural productivity, sustainability, and food security. Continued research and collecting more images for training models and development efforts in AI-driven disease detection have the potential to revolutionize agricultural practices, reduce crop losses, and enhance the resilience of agricultural ecosystems in the face of evolving challenges.

## Data Availability

The datasets analyzed during the current study are available from the corresponding author upon reasonablerequest.

## References

[1] Cheshkova, A. F. A review of hyperspectral image analysis techniques for plant disease detection and identification. *Vavilov J. Genet. Breed.* **26** (2), 202 (2022). 10.18699/VJGB-22-25 PMC8983301 35434482

[2] Munawar, S. M., Rajendiran, D. & Sabjan, K. B. Plant Disease Diagnosis with Artificial Intelligence (AI). *In Microbial Data Intelligence and Computational Techniques for Sustainable Computing*. Singapore: Springer Nature Singapore. 187–193 (2024).

[3] González-Rodríguez, V. E. et al. Artificial intelligence: A promising tool for application in phytopathology. *Hortic* **10** (3), 197 (2024).

[4] Mohammadpoor, M., Nooghabi, M. G. & Ahmedi, Z. An intelligent technique for grape fanleaf virus detection. *Int. J. Interact. Multimed Artif. Intell.* **6** (1), 62–67 (2020).

[5] Khamparia, A. (ed). Microbial Data Intelligence and Computational Techniques for Sustainable Computing. *Springer Nature*. **47** (2024).

[6] Tammina, M. R. et al. Prediction of Plant Disease Using Artificial Intelligence. *Microbial Data Intelligence and Computational Techniques for Sustainable Computing*. Singapore: Springer Nature Singapore. 25–48 (2024).

[7] Gruetzemacher, R. & Whittlestone, J. The transformative potential of artificial intelligence. *Futures* **135**, 102884 (2022).

[8] Pal, S. A paradigm shifts in research: exploring the intersection of artificial intelligence and research methodology. *Int. J. Innovative Res. Eng. Multidisciplinary Phys. Sci.* **11**(3) (2023).

[9] Celeghin, A. et al. Convolutional neural networks for vision neuroscience: significance, developments, and outstanding issues. *Front. Comput. Neurosci.* **17**, 1153572 (2023). 37485400 10.3389/fncom.2023.1153572 PMC10359983

[10] LeCun, Y., Bottou, L., Bengio, Y. & Haffner, P. Gradient-based learning applied to document recognition. *Proc. IEEE*. **86** (11), 2278–2324 (1998).

[11] Ramcharan, A. et al. Deep learning for image-based cassava disease detection. *Front. Plant. Sci.* **8**, 1852 (2017). 29163582 10.3389/fpls.2017.01852 PMC5663696

[12] Arinichev, I. V. Using digital intelligent technologies for the diagnosis of cereals diseases in the Kuban. *Agrar. Sci.* **5**, 70–73 (2022).

[13] Salari, M. et al. Evaluating the application of machine learning in predicting the mortality of hospitalized COVID-19 patients using the confusion matrix and the Matthews correlation coefficient. *Archives Clin. Infect. Dis.* **20**(2), e150150 (2025).

